# Identification of potential candidate genes for lip and oral cavity cancer using network analysis

**DOI:** 10.5808/gi.20062

**Published:** 2021-03-15

**Authors:** Sarmilah Mathavan, Chin Siang Kue, Suresh Kumar

**Affiliations:** Faculty of Health and Life Sciences, Management and Science University, Shah Alam 40100, Malaysia

**Keywords:** biomarkers, carcinogenesis, lip neoplasms, mouth neoplasms, prognosis

## Abstract

Lip and oral cavity cancer, which can occur in any part of the mouth, is the 11th most common type of cancer worldwide. The major obstacles to patients’ survival are the poor prognosis, lack of specific biomarkers, and expensive therapeutic alternatives. This study aimed to identify the main genes and pathways associated with lip and oral cavity carcinoma using network analysis and to analyze its molecular mechanism and prognostic significance further. In this study, 472 genes causing lip and oral cavity carcinoma were retrieved from the DisGeNET database. A protein-protein interaction network was developed for network analysis using the STRING database. *VEGFA*, *IL6*, *MAPK3*, *INS*, *TNF*, *MAPK8*, *MMP9*, *CXCL8*, *EGF*, and *PTGS2* were recognized as network hub genes using the maximum clique centrality algorithm available in cytoHubba, and nine potential drug candidates (ranibizumab, siltuximab, sulindac, pomalidomide, dexrazoxane, endostatin, pamidronic acid, cetuximab, and apricoxib) for lip and oral cavity cancer were identified from the DGIdb database. Gene enrichment analysis was also performed to identify the gene ontology categorization of cellular components, biological processes, molecular functions, and biological pathways. The genes identified in this study could furnish a new understanding of the underlying molecular mechanisms of carcinogenesis and provide more reliable biomarkers for early diagnosis, prognostication, and treatment of lip and oral cavity cancer.

## Introduction

Human head and neck cancers begin in the mouth, nose, throat, larynx, sinuses, or salivary glands. Lip and oral cancer is a subgroup of head and neck cancers that cause lip or oral carcinoma. Oral cancers are often referred to as those occurring in a particular anatomical region, including the lip, gum, tongue, mouth (including the floor of the mouth), and palate, corresponding to the International Classification of Diseases, 10th revision C00-06 code. The vast majority of these cancers (up to 85%–95%) are squamous cell carcinomas, often resulting from pre-existing precancerous lesions. Oral cancer stands out among head and neck tumors due to its frequent occurrence and mortality rate, as well as its common association with a late diagnosis [1].

The low survival rate of oral cancer can be significantly increased if it is detected early or in the pre-cancer stage. Most oral carcinomas are squamous cell carcinomas of the tongue, buccal mucosa, or gums. Lip cancer is the most common tumor in the head and neck of the body, and constitutes 25%‒30% of all mouth cancers [2]. Lip carcinomas are usually basal or squamous cell carcinomas [3]. The oral cavity begins from the blood-red boundary of the lips and extends to the circumvallate papillae of the tongue and the intersection of the soft and hard palate. The oral cavity is divided into the lip, oral tongue, mouth floor, buccal mucosa, upper and lower gum, retromolar trigon, and hard palate [4]. Benign oral cavity lesions include those affecting the anterior tongue, mouth floor, buccal mucosa, retromolar trigone, hard palate, and gingiva.

An estimated 200,000 cases of oral cancer and 100,000 deaths occur every year worldwide. The worldwide age-standardized prevalence of lip cancer was reported to be 0.3 per 100,000 in 2012 (0.4 in males and 0.2 in females) [5]. Smoking tobacco and excessive alcohol intake account for 75% of cases of lip cancer and oral cancer. Other risk factors include chewing of betel juice (*paan*) with or without tobacco and the consumption of nitrosamine-rich foods and salted fish. Another significant risk indicator for oral cancer is the overconsumption of cigarettes [6]. The onset of intake, period, and extent of regular use of chewing tobacco or use of *bidis* as a form of smoked tobacco were closely associated with oral cancer [7]. Smoked tobacco is a major contributor to carcinogenesis in the upper airway, and a positive association exists between tobacco smoke consumption and oral cavity cancer, as documented in numerous studies. The risk of oral cancer is 1.4‒1.7 times higher in those who consume tobacco than in those who do not consume tobacco [8]. *Paan* includes areca nut, betel leaves, and slaked lime, sometimes mixed with tobacco, and certain items like spices, sweets, and essences may be added to *paan*, depending mostly on taste. In Asia, smoking *paan* with or without tobacco is a major risk factor for oral cancer [9], but owing to a lack of knowledge and literacy, many people who routinely use *paan* are unaware of its adverse consequences for health [8].

Other predictors of oral cancer are environmental pollutants such as ultraviolet radiation (lip cancer) or nutritional intake deficits such as fruit and non-starchy vegetables (oral cavity cancer) [5]. Various mutations and genetic mechanisms have also been reported to contribute to lip and oral cancer development and growth. Surgery is usually performed to treat oral squamous cell carcinoma. Surgery facilitates an accurate assessment of the anatomical location, margins, invasive status, and histopathological characteristics of the tumor, and the corresponding advantages and disadvantages may determine the strategy.

To date, the ability to treat advanced oral cancer has been constrained by a lack of understanding of the specific key genes that underlie the growth of this cancer. The aim of this study was to identify key genes through a gene enrichment analysis of lip and oral cavity carcinoma using bioinformatics and to identify novel potential diagnostic biomarkers for oral carcinoma.

## Methods

### Retrieval of disease genes

The genes associated with lip and oral cavity carcinoma (C0220641) were retrieved from the DisGeNET database (accessed December 2019) available at http://www.disgenet.org/home/. DisGeNET is a wide-ranging platform that integrates genes and variants involved in human disease [10]. A total of 472 disease-associated genes were obtained and downloaded for further analysis.

### Network analysis in Cytoscape

The UniProt IDs of associated disease genes recorded from the summary of disease-gene associations were uploaded using the STRING protein query in Cytoscape 3.7.2, which is a precomputed global resource designed to evaluate protein-protein interaction (PPI) information [11]. The confidence score (cutoff) was set to 0.4 and the maximum additional interactors remained the default parameter to obtain more closely related genes to the targeted protein. The STRING network of PPI was constructed and displayed a hierarchical layout for a better view.

### Hub gene retrieval in cytoHubba

The cytoHubba taskbar is a convenient tool for extracting a subnetwork that contains hub genes from an entire large PPI collection. As the scoring method, maximum clique centrality (MCC) was selected to identify featured nodes. In the cytoHubba plugin, the MCC algorithm has been reported to be the most effective method of finding hub nodes. In this study, the top 10 genes with the highest MCC values were considered as hub genes [12].

### Functional and pathway enrichment analysis of hub genes

The WEB-based GEne SeT AnaLysis Toolkit (WebGestalt) (functional enrichment analysis web) integrates management, data retrieval, organization, visualization, and statistical studies of functional enrichment and data visualization, as well as the analysis of large gene sets, and is available at http://www.webgestalt.org/ [13]. The WebGestalt database is unique in that it includes information from various biological contexts, including gene ontology (GO), the Reactome pathway, network module, gene-phenotype and gene-disease interactions, gene-drug associations, and chromosome position. WebGestalt has greatly expanded the scope of functional domains, contributing to a total of 78,612 functional categories. It provides a graphical representation of the data with over-representation enrichment analysis, mainly involving cellular components, biological processes, molecular functions, and biological pathways [14]. A false discovery rate (FDR) ≤ 0.05 was considered to indicate statistical significance.

### Identification of drug candidates based on hub genes

The online tool DGIdb (http://www.dgidb.org/), an available resource containing drug-gene interaction data from more than 30 databases, was used to screen antineoplastic drugs targeting hub genes.

## Results and Discussion

**Fig. 1.**
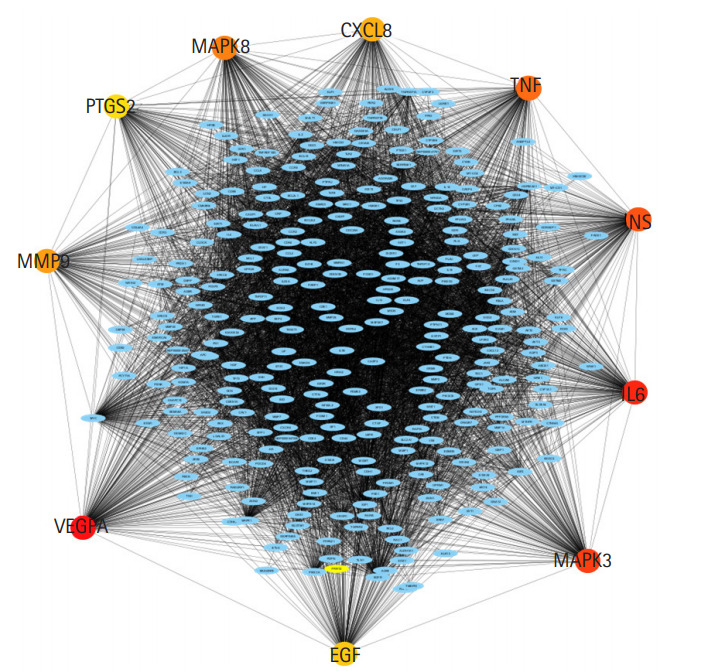
Protein-protein interaction network overview built using STRING in Cytoscape. The network consists of 8,573 edges (interactions) between 444 nodes based on a confidence score of 0.4 and the maximum additional interactors default parameter. Nodes represent proteins, edges represent the interaction between two nodes (proteins).

In total, 472 genes associated with lip and oral cavity carcinoma (C0220641) were identified using the DisGeNET database (Supplementary Table 1). DisGeNET combines text-mined databases with expert-curated databases, provides one of the most extensive sets of associations of human genetic diseases, and is a valuable module for the study of molecular mechanisms underlying genetic diseases [10]. The PPI network was constructed using all 472 genes. PPI analyses help to explain protein roles at the molecular level and to discover the process of cell regulation. The STRING database is often used to evaluate and pre-calculate global-view protein associations comprising 89 full genome sequence datasets, including 261,033 orthologous genes [15]. The visual representation of the predicted, ranked protein interactions network in the STRING database offers a high-level overview of functional associations, enabling extensible analyses of biological systems [15]. In total, 444 nodes and 8,573 edges (interactions) were identified with the STRING network based on a confidence score of 0.4 and the maximum additional interactors as the default parameter (Fig. 1).

**Fig. 2.**
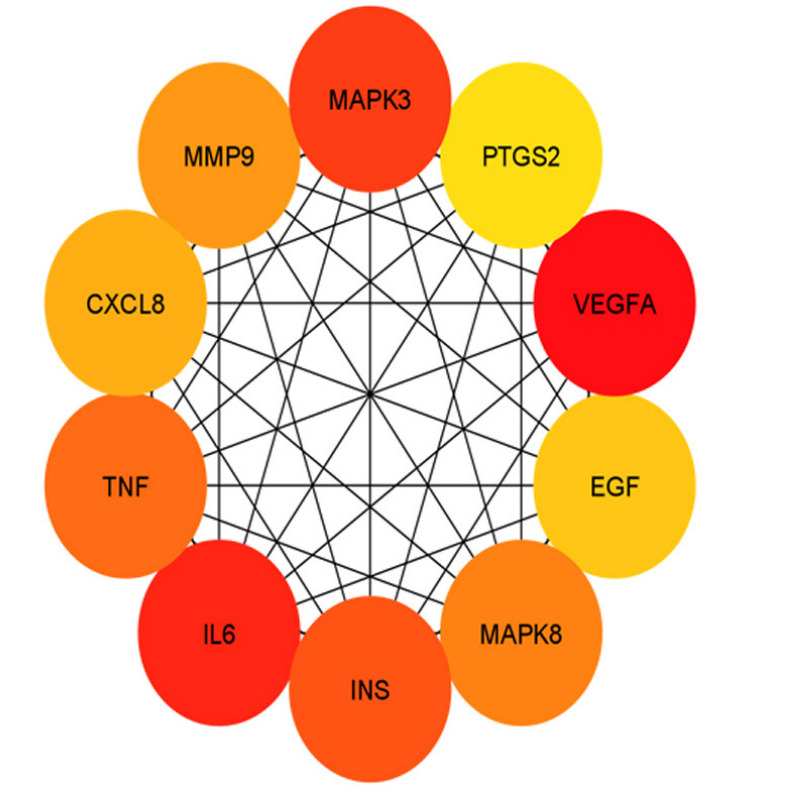
The protein-protein interaction subnetwork consisting of 10 hub genes based on the maximum clique centrality scoring method ranking. The network of the 10 hub genes is shown with red (high ranking) and yellow nodes (low ranking) based on the ranking score.

Nodes and edges are particularly important because they can be linked to data on gene expression and protein structure information. CytoHubba, which has been widely used to explore important nodes in biological networks, was then applied to identify the lip and oral cavity carcinoma hub proteins in the PPI network. First, scores for all 11 methods are given to each node across the pre-loaded PPI network by selecting the options for cytoHubba from the network panel. The MCC score method, which is a local-based method, was chosen for this study. The PPI network and hub genes were visualized using a hierarchical layout. A local rank approach will only recognize the relationship between the node and its significant neighbors to measure the node's score within the network. The MCC method was used to explore the features of the nodes to enhance their efficacy. According to the MCC sores, the top 10 ranked nodes for each specific score system were then obtained from the cytoHubba (Table 1) column in the Cytoscape taskbar and shown in the results column, and the subgraph of all the identified nodes can be seen in the taskbar windows with a large (red) to basic (yellow) color palette (Fig. 2). The top 10 genes associated with lip and oral cavity carcinoma according to MCC scores from the cytoHubba plugin in Cytoscape were screened [12]. The identified key genes responsible for lip and oral cavity carcinoma were vascular endothelial growth factor A (*VEGFA*), followed by interleukin-6 (*IL6*), mitogen-activated protein kinase 3 (*MAPK3*), insulin (*INS*), tumor necrosis factor (*TNF*), mitogen-activated protein kinase 8 (*MAPK8*), matrix metalloproteinase-9 (*MMP9*), interleukin-8 (*CXCL8*), pro-epidermal growth factor (*EGF*), and prostaglandin G/H synthase 2 (*PTGS2*).

The top 10 genes in the string network ranked by the MCC method were then analyzed for gene enrichment analysis using WebGestalt. To identify the functions, a gene enrichment analysis of the hub genes was performed, and an FDR ≤ 0.05 was set as the cutoff value [13].

**Fig. 3.**
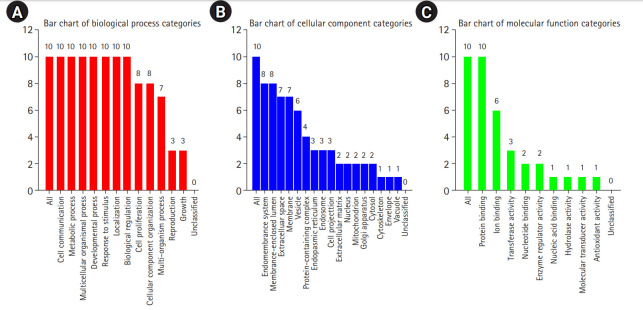
Gene enrichment analysis of 10 significant hub genes (false discovery rate ≤ 0.05) based on the gene ontology slim summary using WebGestalt. (A) Gene enrichment analysis of 10 recognized hub genes based on biological processes. (B) Gene enrichment analysis of 10 recognized hub genes based on cellular components. (C) Gene enrichment analysis of 10 recognized hub genes based on molecular function.

**Fig. 4.**
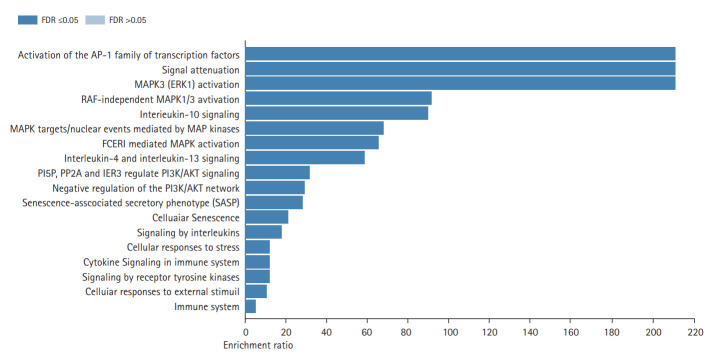
Gene enrichment analysis of 10 recognized significant hub genes (false discovery rate [FDR] ≤ 0.05) based on the biological pathways of the Reactome database.

Fig. 3B shows the results of the cellular component GO term enrichment analysis, which suggested that all 10 hub genes were significantly enriched in the endomembrane system. The genes are also present in the membrane-enclosed lumen, extracellular space, membrane, and vesicle, and actively function in the protein-containing complex, endoplasmic reticulum, endosome, cell projection, and extracellular matrix. Other cellular components involving these hub genes include the nucleus, mitochondrion, Golgi apparatus, cytosol, cytoskeleton, cell envelope, and vacuole. The cell polarity position of the endomembrane pool of Cdc42 and the possible role of this pool in cancer-related alterations are known. The Golgi apparatus was noted to be rapidly oriented towards the posterior end of the plasma membrane, meaning that its integrity is necessary for guided cell motility and polarized secretion [16]. In the form of single-strand malfunctions, tobacco smoking causes significant damage to DNA [17], and increases in protein thiols and lipid peroxidation/oxidation [18]. Mitochondrial DNA is vulnerable to harm from reactive oxygen species such as superoxide radicals, hydrogen peroxide, and hydroxyl radical due to a lack of a defensive histone backbone [19]. In the vast system of close and distant cell-to-cell communication, extracellular vesicles (EVs) are secreted by most cell types [20]. Under the influence of hypoxia-inducible factors, EVs that developed under a stressful environment showed enhanced proliferation and migration of oral cancer cells [21]. As for the molecular function of the lip and oral cavity carcinoma hub genes (Fig. 3C), most of these genes are involved in protein binding, with moderate functions in ion binding, transferase activity, nucleotide binding, and enzyme regulatory activity. Protein binding is a secreted glycoprotein that enhances cell-cell and cell-extracellular matrix permeability and induces the production of IL-1, IL-6, and other blood monocyte cytokines, contributing to the invasion and metastasis of lip and oral cancer cells [22]. Antioxidant disruption to salivary DNA and proteins can encourage oral squamous cell carcinoma [23]. The mechanism through which oxidative damage is involved in oral cancer is that when the salivary DNA is extracted from exfoliated oral epithelium, the oxidized proteins and DNA contained in the saliva interact with salivary free radicals [24]. Hydrolase is involved in the metabolism of tobacco carcinogens [25]. Hydrolase activity is linked to an increased risk of oral cavity, pharyngeal, and laryngeal cancers, which are smoking-related cancers, and recent study supported its involvement in lung cancer [26]. In terms of biological processes (Fig. 3A), most of the hub genes are involved in cell communication, metabolic processes, multicellular organismal processes, developmental processes, responses to stimuli, and localization and biological regulation. These hub genes are also involved in cell proliferation, cellular component organization, and multi-organism processes. Cancer cells, which have altered glucose and lipid metabolism, show radical improvements in energy metabolism function compared to normal cells. Tumor metabolism studies have reported that oncogenic signaling pathways stimulated metabolic reprogramming to upregulate lipid, carbohydrate, protein, DNA, and RNA biosynthesis, leading to enhanced tumor development. Under aerobic conditions, cancer tissues have elevated glycolysis levels in the cytosol, even with fully functioning mitochondria, as a result of phosphoinositide 3-kinase (PI3K)/AKT signaling; this is known as the Warburg effect [27]. The cell's regular activities and organization are closely regulated by excitatory or inhibitory input [28]. In tumor cells, pathways are altered, enabling them to divide quickly, sequester blood vessels that stimulate growth, remove or enhance signals to create abnormal functional or structural alterations, and penetrate local or remote sites of normal tissue [29]. Fig. 4 shows the biological pathways (Reactome pathways). Most of these genes are involved in activation of the AD-1 family of transcription factors, signal attenuation, and MAPK3 (ERK1) activation. The transcription factor protein-1 (AP-1) superfamily activator is known to modulate gene expression during the development of many cancers and has been recognized as a potential target for modern therapeutic applications. Its components are involved in RAF-independent MAPK 1/3 activation, interleukin-10 signaling, MAPK targets/nuclear events mediated by MAPKs and high-affinity IgE receptor (FCERI)-mediated MAPK activation including interleukin-4 and interleukin-3 signaling. These hub genes were found to be involved in phosphatidylinositol-5-phosphate/phosphatidylinositol-4,5-bisphosphate and immediate early response 3-regulated regulate phosphatidylinositol 3-kinase and Akt/Protein Kinase B (PI3K/AKT) signaling, regulation of PI3K/AKT signaling, senescence-associated secretory phenotype, cellular senescence, and signaling by interleukin. The PI3K/Akt pathway is a central controller of viability in response to cell stress (e.g., pH, nutrient, and oxygen levels), and deregulation of the PI3K signaling pathway leads to cancer [30].

*VEGFA* encodes a platelet-derived growth factor/vascular endothelial growth factor (VEGF) family member that acts as a glycosylated mitogen, increasing endothelial permeability, angiogenesis, vasculogenesis, endothelial cell growth, and cell migration. VEGFA functions as a central stimulator of angiogenesis, which is an important trait of cancer that plays a crucial role in tumor growth. The production of VEGFA is induced by the generation of hypoxic conditions within tumors [31]. A study reported that VEGFA mRNA levels were 53-fold higher in oral carcinoma tissues than in normal tissues. Hence, VEGFA functions as a potent autocrine survival factor for cancer cells. The risk of oral cancer may be correlated with VEGFA locus haplotypes, and the haplotype effect may be more substantial than a single susceptibility polymorphism [32]. Compared to the normal oral mucosa, multiple studies have shown upregulation of VEGFA expression in cancerous tissues. VEGF levels in oral cancer patients were also found to be significantly higher than normal controls, in an analysis that included clinical stage and lymph node metastasis. This suggests that VEGFA levels may be a reliable biomarker and that VEGFA may be a potential target for developing chemotherapy strategies for oral carcinoma patients.

IL-6 is a pleomorphic cytokine involved in various physiological and pathological processes, such as responses to trauma and infection and the progression of inflammation and tumors. IL-6 appears to lead to oral cancer pathogenesis via multiple pathways and biological processes [33]. IL-6 can stimulate the release of matrix metalloproteinase 1 and 9, which are responsible for malignant growth and neoangiogenesis in oral squamous cell carcinoma. Several keratinocyte mechanisms, including cell formation, survival, and differentiation, are also modulated by IL-6. By triggering global hypomethylation and changes in DNA methylation trends in oral cancer cells, IL-6 can contribute to the growth of oral cancer [34]. IL-6 is linked with increased tumor growth and metastasis, and may therefore be involved in this disease's pathogenesis. Serum IL-6 was detected at higher concentrations than salivary IL-6 in oral cancer patients. Therefore, serum IL-6 was proposed as a diagnostic or prognostic marker for oral cancer and pre-cancer.

Tumor cell development, differentiation, apoptosis, angiogenesis, invasion, and metastasis are associated with MAPK3 and MAPK8. The repression of MAPK signaling caused by irregular gene expression leads to abnormal responses, whereas regulated MAPK inhibits the development of inhibitory proteins in cell cycles. PI3K/Akt gene mutations lead to irregular activation of the MAPK pathway in oral cancer. This observation suggests an intricate relationship between the MAPK and PI3K/Akt pathways [35].

*INS* encodes insulin. By modifying the insulin receptor substrate-1 (IRS-1) pathway, diabetes can also raise the risk of certain forms of cancer, including oral carcinoma. Changes in pathway are thus an intermediate step towards neoplasia, involving cytoskeleton modifications and decreased cell adhesion. Both integrin and focal adhesion kinase involvement induce IRS-1 activation upon *INS* activation by tyrosine phosphorylation [36]. TNF promotes cell proliferation and apoptosis, and was reported to be present at high levels in patients with oral leukoplakia, oral lichen planus, and oral submucous fibrosis, which have been claimed to be clinical biomarker for oral cancer [37].

MMP-9 is also a possible biomarker of oral cancer. MMP-9 is a family of enzymes that has been found to be linked to tumor progression because they are active in extracellular matrix breakdown. More specifically, MMP-9 is a family of zinc-dependent proteinases associated with type IV collagen, a key source of the basal lamina, and other forms of collagen in various pathological conditions [38].

CXCL8, also known as interleukin-8 (IL-8), is involved in oral cancer invasiveness through activation of MMPs. Metastatic activity was correlated with interactions between IL-8 and MMP. Well-established inflammatory cytokines in oral cancer cells have been shown to affect MMP development [39].

The EGF receptor is a cell-surface tyrosine kinase known to regulate the metastasis and recurrence of oral cancer. Abnormal stimulation of the downstream signaling pathways promotes the epithelial-to-mesenchymal transition, which ultimately results in neoplastic cells with elevated invasive and metastatic capability [40].

In oral cancers, PTGS2 or cyclooxygenase 2 was elevated. Manifesting as broad hypomethylation in the promoter region of the CpG island area, PTGS2 expression occurs at various organ sites in response to stress, cigar smoke, and pharmacological drugs, altering the methylation of the PTGS2 promoter [41].

Based on the 10 predicted hub genes as potential therapeutic targets for lip and oral cancer, we identified several antineoplastic drugs based on the DGIdb database. Specifically, nine small-molecule drugs (ranibizumab, siltuximab, sulindac, pomalidomide, dexrazoxane, endostatin, pamidronic acid, cetuximab, and apricoxib) were identified as potentially having therapeutic effects for lip and oral cavity cancer based on their interaction scores in the DGIdb database. The interaction scores were calculated based on the evidence score and relative drug and gene specificity. Table 2 shows small-molecule drugs with potential therapeutic effects for lip and oral cavity cancer based on the highest interaction score for each predicted hub gene. However, it is indeed necessary to support promising therapeutic targets with more studies.

Our analysis has many advantages over previous work [42-44]. First, this study had a broad sample size retrieved from the DisGeNET database, which covers research from expert-curated repositories, genome-wide association study catalogs, animal models, and scientific literature. We further studied the functional and pathway enrichment of important genes. To our best knowledge, we identified some heretofore unreported prognostic biomarkers, such as *MAPK3*, *INS*, and *PTGS2*. However, further clinical validation of these reported biomarkers is needed.

## Conclusion

A comprehensive perspective was provided by the bioinformatics analysis to understand the mechanism underlying lip and oral cavity carcinoma development. In this study, the following hub genes were identified as being involved in lip and oral cavity carcinoma through network analysis: *VEGFA*, *IL6*, *MAPK3*, *INS*, *TNF*, *MAPK8*, *MMP9*, *CXCL8*, *EGF*, and *PTGS2*. In total, 472 gene-disease associations and 10 hub genes were identified and recognized as target biomarkers for lip and oral cavity carcinoma. We also identified several antineoplastic drugs with potential applications for lip and oral cavity cancer. A detailed study of the genes’ biological mechanisms and their pathways may provide potential targets for the therapeutic drug monitoring of lip and oral cavity carcinoma. Nevertheless, further studies are required to understand the development of lip and oral cavity carcinoma to unravel its mechanism more completely.

## Figures and Tables

**Table 1.** The top 10 ranked nodes were selected using the MCC method in the cytoHubba app in Cytoscape 3.7.2

Rank	Gene name	MCC score
1	*VEGFA*^a^	1.88148120178673E+37
2	*IL6*	1.88148120178002E+37
3	*MAPK3*	1.88148120172653E+37
4	*INS*	1.88148118653989E+37
5	*TNF*	1.88148116290353E+37
6	*MAPK8*	1.88148112775536E+37
7	*MMP9*	1.88147822258162E+37
8	*CXCL8*	1.88147770682472E+37
9	*EGF*	1.88147075212715E+37
10	*PTGS2*	1.88128391642781E+37

These top 10 ranked nodes represent the top 10 hub genes of lip and oral cavity carcinoma.

a Based on these results, *VEGFA* was identified as the highest-ranked hub gene with the highest maximum clique centrality (MCC) score.

**Table 2.** Antineoplastic drugs targeting the predicted hub genes for lip and oral cavity cancer based on the DGIdb database

Hub gene	Drug	Type	Source	Interaction score
VEGFA	Ranibizumab	Inhibitor	DrugBank, TdgClinicalTrial, ChemblInteractions, TEND, PharmGKB TTD	6.51
IL6	Siltuximab	Inhibitor	DrugBank, MyCancerGenome, ChemblInteractions, TTD	8.61
MAPK3	Sulindac	Inhibitor	DrugBank	0.34
INS	-	-	-	No interaction
TNF	Pomalidomide	Inhibitor	DrugBank	0.22
MAPK8	Dexrazoxane	-	NCI	0.39
MMP9	Endostatin	-	DrugBank	0.82
CXCL8	Pamidronic acid		NCI	0.39
EGF	Cetuximab		CIViC, PharmGKB	
				1.16

PTGS2	Apricoxib	Inhibitor	TALC, TdgClinicalTrial, ChemblInteractions	1.39

The interaction score was calculated from the DGIdb database based on the evidence score and relative drug and gene specificity. The table shows small-molecule drugs with potential therapeutic effects for lip and oral cavity cancer based on the highest interaction score for each predicted hub gene.

## Supplementary Materials

Supplementary Table 1. A total of 472 genes associated with lip and oral cavity carcinoma (C0220641) obtained from the DisGeNET database
