# Supplementary material for: Identification of potential candidate genes for lip and oral cavity cancer using network analysis
Source: Genomics Inform. 2021 Mar 15;19(1):e4. doi: 10.5808/gi.20062 (PMC8042300; doi:10.5808/gi.20062)
Supplement: Supplementary Table 1. — A total of 472 genes associated with lip and oral cavity carcinoma (C0220641) obtained from the DisGeNET database [file gi-20062-suppl.pdf]

Supplementary Table 1. A total of 472 genes associated with lip and oral cavity carcinoma (C0220641) obtained from the DisGeNET database

Disease name	Disease-associated gene symbols
Lip and oral cavity carcinoma	P49789, P08253, O75179, Q8IWV2, Q13884, Q8IV61, Q70Z35, P04637, Q9UPU9, P35354, P30711, P09488, P42771, Q8N726, Q13002, P24385, P00533, P15692, P04798, O60760, Q7Z6W7, P00403, P42336, P01375, P61073, Q00987, P10145, P12004, P18887, P19838, Q8TCG1, P08183, P42338, P10415, O95980, P48736, O00329, P31749, O15350, Q8N264, Q12999, Q9HD26, P05181, Q9Y6X3, P09211, P09601, P13501, P48061, P07858, Q8WUP2, Q07820, P42898, P11245, Q9NR45, P27361, Q86UG4, P04626, Q9Y2Q3, P42574, Q92574, P01137, P37173, P01133, P01135, O14746, Q14517, P18074, Q15910, P04179, P28161, P10826, P09237, Q8WV07, P16455, P22301, P05231, Q6ZT07, P05362, P60484, P14780, Q9UPY3, P03973, Q16665, P24347, P51681, P16070, P12830, P00326, Q16790, Q06124, P05112, O60341, O00409, P25963, O14519, P01344, P11926, P05121, Q96EB6, Q9UQ07, P01112, Q9H4A3, Q04206, O75838, P46527, O94907, Q14116, P04040, Q03135, Q9UM13, P54368, O60936, P36952, P40692, P00749, P05161, P06748, Q92879, P28482, Q9Y251, Q9UDY8, Q4L180, Q6UXR4, P27701, P12821, Q9NR96, P00352, P29323, P08047, Q9HCY8, Q12778, P24522, P14921, P43268, Q08050, Q15109, P13500, P21266, P21579, Q15672, Q9H3H5, P49767, P29279, P14210, Q9NZW4, P25063, Q13427, O95999, Q13501, O15055, Q9UBH6, Q6ZQN5, P41597, Q9UQ84, O60882, O43474, O95832, P02786, P35442, Q9UBB5, Q6ZN17, O43490, Q9UBN6, P19438, P20333, Q92187, P13010, Q13426, P55055, O43543, O14980, P08670, P04628, A8MW95, P50591, P52888, P29401, Q14457, Q9H3D4, O14788, Q9Y2T1, Q9Y490, O15169, Q01650, O60603, P28289, P23381, Q53EZ4, Q9NPA2, Q9GZV8, Q8N5D6, O60381, Q96PG8, Q9BXH1, P35226, Q53EL6, Q6XE24, Q9BQQ3, Q9UKR3, Q9H488, Q9UQ35, Q9UBY5, Q9UHK6, Q9UIK5, O95239, Q6ISB3, Q8NF37, Q9H2E6, P38159, Q9NPF7, Q9NZC7, P57735, Q9BXP2, Q9NRX1, Q9NYB0, Q8WUY9, Q99612, Q9UHB6, Q9GZX7, Q9Y5C1, Q9UJ41, Q96HU1, Q9BWT7, Q9UHY1, Q9BZS1, Q9BRK5, Q6U949, Q9NTG7, Q99618, P19883, P31941, O60645, Q5VWK5, Q96CG8, Q99985, Q8WWM9, Q9UJZ1, O00165, Q92597, O15516, Q13671, O60315, Q9Y243, P61289, P14550, Q9UGM6, Q9Y5W3, Q7Z6L0, P83110, O75935, Q8WV24, Q13201, Q5TBK1, Q9BXY0, Q2M1K9, Q9BRT3, Q8WYH8, Q9UJ72, Q9Y5W5,

	Q99828, O95994, Q86YL7, Q8N3U4, Q9UGL1, Q8NHL6, Q14201, Q9NQ30, P56945, Q16635, P35221, P30740, Q15717, Q14247, P0DP91, Q03468, P11308, P13726, Q13642, P02751, P24530, Q9H8V3, P15924, P35222, P07711, P11509, P20813, Q9UI36, P07585, P35638, P10515, Q9UBC3, P42345, P19526, P09429, P54868, P31269, P31260, P00738, Q00613, P34932, O00219, P08263, P00390, Q10981, P12956, P10253, Q06546, P32455, P55107, Q03113, P07203, P41594, P07900, Q15828, P18440, P10275, Q13315, P01185, Q07812, Q07817, Q92843, P20749, P55957, P35226, Q92482, P05067, O15392, P16442, P35318, P29275, P09874, P31751, Q13740, P09917, P08758, P25054, P22003, P15056, P49918, Q12798, P32246, P51677, P51684, Q01955, Q96IY4, P02741, P02511, P38936, P11802, P30304, P29466, P49662, P55210, Q16589, P20963, P16671, P34810, P06493, Q12834, Q16539, P01210, P23219, Q12913, P23471, P63000, O75943, O60216, P62834, Q13972, P07949, O15151, P26599, O60542, Q03405, P00747, P53350, Q07869, P37231, Q15172, Q16659, P45983, O14733, P08922, P28702, P11166, P31645, P28370, P51532, Q07889, P48431, P10451, P42224, O14965, Q13309, Q15465, Q13326, P23297, P29034, P26447, P06702, P04271, P53778, P13236, Q8NHW4, Q96HF1, P78536, P49585, P24821, P41159, P48357, P09382, Q08380, P15018, P49917, O60427, P48449, Q08477, P80188, Q14847, P13646, P05019, P11717, P01584, P60568, P35225, P01308, O60674, P52732, P13647, P09758, P84022, Q16236, O00746, P40261, Q9UM47, P29728, O15527, P35372, Q06830, Q13153, Q15843, P12524, P01106, Q13485, P03956, P50281, P22897, O43795, P43246, P00395, P48039, P53602, Q15366

The gene symbol represents the UniProt ID since DisGeNET is an integrated database.
